# Isolation, Whole-Genome Sequencing, and Annotation of Three Unclassified Antibiotic-Producing Bacteria, Enterobacter sp. Strain RIT 637, Pseudomonas sp. Strain RIT 778, and *Deinococcus* sp. Strain RIT 780

**DOI:** 10.1128/MRA.00863-21

**Published:** 2021-12-02

**Authors:** Marissa N. Schroeter, Safiya J. Gazali, Anutthaman Parthasarathy, Crista B. Wadsworth, Renata Rezende Miranda, Bolaji N. Thomas, André O. Hudson

**Affiliations:** a The Gosnell School of Life Sciences, Rochester Institute of Technology, Rochester, New York, USA; b College of Health Science and Technology, Rochester Institute of Technology, Rochester, New York, USA; Loyola University Chicago

## Abstract

We report the isolation, whole-genome sequencing, and annotation of Enterobacter sp. strain RIT 637, Pseudomonas sp. strain RIT 778, and *Deinococcus* sp. strain RIT 780. Disk diffusion assays using spent medium demonstrated that all bacteria produced bactericidal compounds against Escherichia coli ATCC 25922, Pseudomonas aeruginosa ATCC 27853, and Staphylococcus aureus ATCC 25923.

## ANNOUNCEMENT

Three bacteria belonging to the genera Enterobacter, Pseudomonas, and *Deinococcus* were isolated for their antimicrobial-producing properties. Enterobacter species are generally considered human pathogens, but some strains have been shown to possess antibacterial (1–3) and antifungal (4) activities. Pseudomonas species also produce antibacterial (5–9) and antifungal compounds (10, 11); their genomes have been mined for the ability to synthesize secondary metabolites and drug-like natural products (12), and P. putida produces a number of natural product families (13). Deinococcus radiodurans produces an antioxidant exopolysaccharide, while a *Deinococcus* strain isolated from ants produces cancer-preventing aminoglycolipids (14).

**FIG 1:**
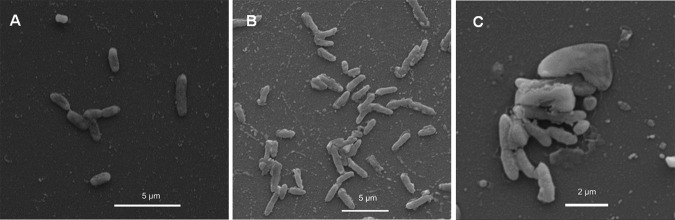
Scanning electron microscopy images of the bacteria: (A) RIT 637 (magnification, 20,000×), (B) RIT 637 (magnification, 14,700×), and (C) a mixed culture of RIT 778 (rods) and RIT 780 (cocci) (magnification, 32,100×).

RIT 637 was isolated from the rhizosphere of the tree Malus sylvestris on Reasoner’s 2A (R2A) medium, and RIT 778 and RIT 780 were both isolated from a water sample (Lake Ontario) on a 1:1 mixture of R2A and LB media. The bacteria are shown in electron microscopy images (Fig. 1) using published methods (5). The inhibition activity of their spent medium extracts against Escherichia coli ATCC 25922, Pseudomonas aeruginosa ATCC 27853, and the Gram-positive strain Staphylococcus aureus ATCC 25923 was verified using disk diffusion assays, according to published methods (5).

The GenElute kit (Sigma-Aldrich, USA) was used according to the manufacturer’s protocol; DNA was quantified using the Qubit 3.0 high-sensitivity (HS) assay and diluted to 0.25 ng/μL. Sequencing libraries were prepared using the Nextera XT kit according to the manufacturer’s specifications (Illumina, Inc., San Diego, CA). Unique dual-indexed libraries were pooled, diluted to 4 nM, and denatured and sequenced using the Illumina MiSeq platform (v3 600-cycle cartridge; paired-end, 2 × 300-bp format). Adapter trimming was conducted using Trimmomatic v0.39 to remove bases with a Phred quality score of <15 over a 4-bp sliding window (15). Reads <36 bp long, or those missing a mate, were removed. SPAdes v3.14.1 was used for *de novo* assembly with default parameters (16). QUAST (http://cab.cc.spbu.ru/quast/) was used for quality assessment, excluding any contigs of <500 bp (17). The genera and species of the genomes were identified using the Type Strain Genome Server (https://tygs.dsmz.de) (18). An assembly could not be assigned to a particular species with <80% sequence identity to the type strains. The completeness and contamination were assessed using CheckM v1.0.18 (19) and determined to be 99.96% and 2.08% for RIT 637, 99.95% and 0.6% for RIT 778, and 99.15% and 0.43% for RIT 780, respectively. The assemblies were submitted to GenBank for annotation of the open reading frames (ORFs), tRNAs, and rRNAs using the Prokaryotic Genome Assembly Pipeline v5.2 (20, 21) (Table 1).

**TABLE 1:** Sequencing and annotation results for Enterobacter sp. strain RIT 637, Pseudomonas sp. strain RIT 778, and *Deinococcus* sp. strain RIT 780

Organism	GenBank accession no.	SRA accession no.	Assembly size (bp)	No. of contigs	No. of raw reads	Coverage (×)	*N*_50_ (bp)	GC content (%)	No. of ORFs	No. of tRNAs	No. of rRNAs
Enterobacter sp. RIT 637	JAIHAZ000000000.1	SRR15447009	4,947,785	230	1.59E+06	97	244,147	55.55	4,736	77	4
Pseudomonas sp. RIT 778	JAIHBA000000000.1	SRR15447008	6,424,674	81	1.91E+06	89	147,603	60.75	5,794	63	4
*Deinococcus* sp. RIT 780	JAIHBB000000000.1	SRR15447007	4,203,250	394	2.09E+06	149	19,530	69.27	3,917	49	3

Antibiotic biosynthetic gene clusters (BGC) were identified using antiSMASH v5.0 (22) and ARTS (23). RIT 637 contains 7 BGC with 1 self-resistance gene; RIT 778 contains 14 antibiotic BGC with one case of self-resistance, while RIT 780 contains 5 BGC, all with no similarity to known BGC. The potential novelty of the secondary metabolites is indicated by the low similarity of 5 of 7 BGC (RIT 637), low/no similarity for 12 of 15 BGC (RIT 778), and the complete lack of similar BGC for all 5 entries (RIT 780).

### Data availability.

The whole-genome assemblies have been deposited in GenBank under the accession numbers JAIHAZ000000000.1, JAIHBA000000000.1, and JAIHBB000000000.1 (Table 1).
